# Trends of overweight and obesity among preschool children from 2013 to 2018: a cross-sectional study in Rhine-Neckar County and the City of Heidelberg, Germany

**DOI:** 10.1186/s12889-022-13302-w

**Published:** 2022-05-11

**Authors:** Weina Liu, Mike Z. He, Peter Dambach, Rainer Schwertz, Simiao Chen, Fengyun Yu, Michael Marx

**Affiliations:** 1 Faculty of Medicine and University Hospital, Heidelberg Institute of Global Health (HIGH), Heidelberg University, Im Neuenheimer Feld 130, Heidelberg, Germany; 2 Jiangsu Province Center for Disease Control and Prevention, Nanjing, China; 3 Department of Environmental Medicine and Public Health, Icahn School of Medicine at Mount Sinai, New York, NY USA; 4 Public Health Authority of Rhein-Neckar-Kreis, Heidelberg, Germany; 5 Chinese Academy of Medical Sciences and Peking Union Medical College, Beijing, China

**Keywords:** Trends of overweight and obesity, Preschool children, Cross-sectional study, Migration background

## Abstract

**Background:**

Early childhood overweight and obesity is a growing public health concern worldwide. Few recent studies have addressed how time trends varied by sociodemographic characteristics at the regional level using large and high-quality data. This study determines how time trends vary in the prevalence of early childhood overweight and obesity by age, gender, and migration background at the regional level.

**Methods:**

We used a Kernel-density curve to describe the BMI distribution, and evaluated the trends of overweight and obesity by age, gender, and migration background using logistic regression.

**Results:**

Mean BMI and the overall prevalence of overweight and obesity increased among preschool children aged 4–6 years in the Rhine-Neckar County and the City of Heidelberg. After adjusting for age, sex, and migration background, trends of overweight significantly increased only among male children in the age 5 year group with migration background (*P* < 0.05), and an upward trend of obesity was observed only among male children in the age 5 year group and female children in the age 6 year group with migration background (*P* < 0.05).

**Conclusions:**

BMI distribution as well as general prevalence of overweight and obesity are still increasing among preschool children. Children with migration backgrounds, particularly male children in the age 5 year groups and female children in the age 6 year group should be prioritized. Health promotion strategies for children with migration backgrounds will help address this challenge.

**Supplementary Information:**

The online version contains supplementary material available at 10.1186/s12889-022-13302-w.

## Introduction

Early childhood is a critical stage of life, marked by significant physical and neural development. Particularly, early childhood overweight and obesity is a growing public health concern worldwide. Early childhood overweight and obesity not only lead to adverse consequences including physical and mental health disorders [1, 2], but also results in impacts on the later adulthood life, such as an increased risk of diabetes, osteoarticular diseases, cardiovascular diseases, and cancer [2]. The pooled prevalence estimates of obesity and overweight in European children (aged 2–7 years) was 22.2% from 2006 to 2016 [3], which was higher than those in Africa (10.4%), North America (12.1%), Asia (5.7%) and Latin America and the Caribbean (7.0%), and other developing countries(7.2%) during same period [4]. According to the results of the German Health Interview and Examination Survey for Children and Adolescents, the prevalence of overweight among preschool children in Germany was 10.8% for female children and 7.3% for male children, and the prevalence of obesity was 3.2% for female children and 1.0% for male children between 2014 and 2017 [5].

Existing studies show that the trend of overweight and obesity in preschool children are changing. A study performed in Bavaria showed an increasing trend from 1982 to 1997 [6]. Another investigation covering all of Germany conducted during 1991 to 2000 also indicated the same trend [7]. In comparison, the trend was decreasing in 16 German federal states from 2004 to 2008 [8]. A similar decreasing trend was also observed in the results of the German Health Interview and Examination Survey for Children and Adolescents between 2014 and 2017 [5]. Although Germany has achieved the WHO target for controlling obesity among children and adolescents, the prevalence of obesity remains at a high level. Overweight and obesity could already lead to lower life satisfaction [9] and more school bullying [10] than that of normal children in early childhood, and these consequences of obesity can carry on into adulthood [11]. Therefore, it is important from a public health perspective to rigorously evaluate the development of overweight and obesity with large and high-quality samples at a regional level, as well as contextualize them internationally. This study is the first to describe the trend of overweight and obesity stratified by socioeconomic characteristics using a dataset involving 37,858 preschool children in Heidelberg. In addition, the up-to-date estimates are essential to fill the gap of studies on the trend of overweight and obesity in Germany before 2018.This study aims to explore how the prevalence of overweight and obesity during early childhood varies by age, sex, and migration background at the regional level over time by means of a large dataset from the School Entry Health Examination (SEHE), which includes all preschool children in the Rhine-Neckar County and the City of Heidelberg, Germany from 2013 to 2018 [12]. This study may provide evidence for local health policymakers at regional levels to implement programs for primary prevention of obesity.

## Methods

### Data sources and study design

According to the laws and regulations in Baden-Württemberg [13–15], all children who reached the calendar age for obligatory school attendance were requested to attend school entry health examination prior to their sixth birthday. Each child in the sample was only measured once between 2013 and 2018. From 2013 to 2018, 37,858 children from 454 kindergartens of 54 district towns and municipalities participated in the SEHE. We conduct a cross-sectional study with data from SEHE during this period, in order to describe how the prevalence of overweight and obesity changed by age, sex and migration background. All children’s data were anonymized before analyses.

### Measurements and covariates

The generated data includes measurements of height and weight and general sociodemographic information (age, sex, and migration background). Children were required to wear light clothes and take off shoes. Body height was accurate to the nearest 1 cm, and body weight was accurate to the nearest 100 g. BMI was classified as underweight, normal weight, overweight, and obese according to BMI-for-age Z-scores from the World Health Organization child growth standards [16]. Immigrant background was defined as children with at least one of their parents being born abroad or the language spoken at home is not German or it is German together with another language, as described by Schenk et al. [17]. If any children whose information such as age, sex, migration background, weight, or height were missing, they were excluded from the study.

### Statistical analysis

We described the distribution change in BMI between 2013 and 2018 using a Kernel-density curve [18, 19], which is a nonparametric smoothed graph. The trend of overweight and obesity was evaluated with the adjustement of age, sex, and migration background using a logistic regression model with the survey year as independent variable (continuous variable) and the statuses of overweight/obesity as dependent variables (binary variables). Model I was adjusted by age and sex. Model II was adjusted by age, sex, immigrant background, and survey year. Statistical significance was set at *P* < 0.05. We used the statistical software R (version 3.6.3) to analyze the data.

## Results

### Characteristics of the study participants

In this survey, 37,858 children aged 4 to 6 years old were enrolled from 2013 to 2018. 33,407 children had valid information, including 17,304 male children and 16,103 female children. The response rate was 88.2%. The percentage of children that had a migration background was 50.8%. The overall prevalence of overweight and obesity was 7.6% and 2.8%, respectively. The children ages 4, 5, and 6 accounted for 12.2%, 75.0% and 12.8% of the population respectively. The baseline characteristics of the study population are shown in Table 1.

### Secular trend in BMI

**Fig. 1:**
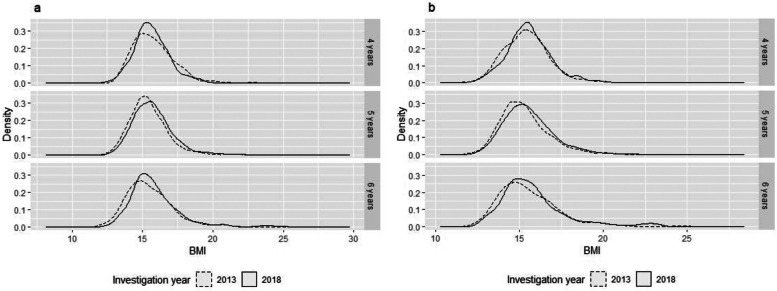
Distribution change of body mass index among male (**a**) and female (**b**) preschool children

From 2013 to 2018, mean BMI increased from 15.5 to 15.7 kg/m^2^ for male children, and increased from 15.4 to 15.6 kg/m^2^ for female children. Similar upward trends were observed among almost all preschool children except for male children aged 4 years (Table S1). The BMI distribution curves for male children and female children all shifted to the right from 2013 to 2018, except for male children of aged 4 years (Fig. 1).

### Trends of overweight

**Fig. 2:**
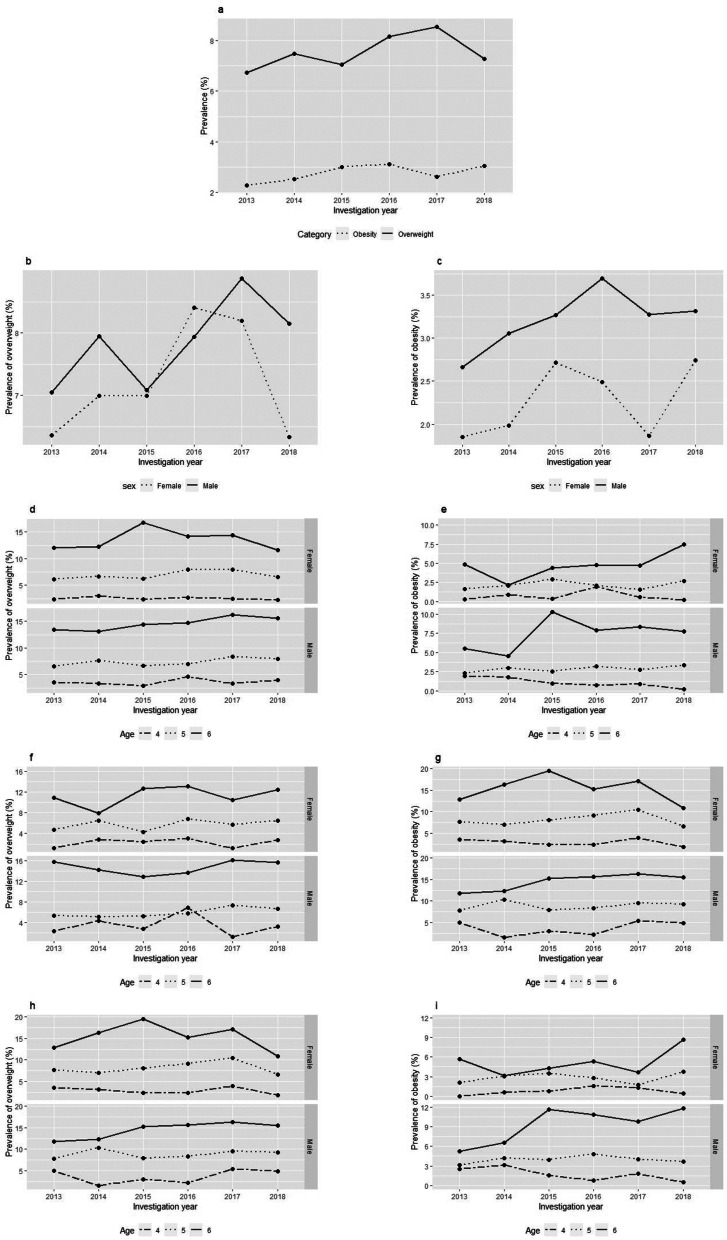
The trend of overweight and obesity from 2013 to 2018. **a **The general trend. **b**,**c** The trend adjusted by sex. **d**,**e** The trend adjusted by sex,age. **f**,**g**,**h**,**i** The trend adjusted by sex,age and migration background

From 2013 to 2018, the prevalence of overweight (6.7% vs7.2%) in preschool children increased significantly in Rhine-Neckar County and the City of Heidelberg (Fig. 2(a)), especially for the 5-year old age group (*P* < 0.05; Table S2). As shown in Table S3 and Table S4, the prevalence of overweight increased from 7.1% to 8.2% for male children, while showing no upward trend for female children (*P* > 0.05). After adjusting by age and sex (Fig. 2(b)), the prevalence of overweight for male children increased from 6.5% to 8.0% among 5-year olds(*P* < 0.05;Table S3; Fig. 2(d)), while for female children it did not significantly increase across all age groups during this period (*P* > 0.05;Table S4; Fig. 2(d)). After adjusting by age, sex, and migration background, for male children, the prevalence of overweight increased from 5.4% to 6.7% among 5-year olds with migration background (*P* < 0.05;Table S3; Fig. 2(f)), while no significant changes were observed for female children(*P* > 0.05;Table S4; Fig. 2(f) and (h)) across all age groups, irrespective of migration background.

### Trends of obesity

When comparing with the trend of overweight, the prevalence of obesity (3.0%, 2018 vs 2.2%, 2013) also increased significantly across all age groups over the 6-year period (*P* < 0.05; Table S5, Fig. 2(a)). After adjusting by sex and age, the prevalence of obesity among male children and female children barely changed from 2013 to 2018 (*P* > 0.05; Table S6 and S7, Fig. 2(c)); however, it increased among 6-year olds in both sex (*P* < 0.05; Table S6 and Table S7, Fig. 2(e)). After adjusting by sex, age, and migration background, male children aged 5 years with migration background and female children aged 6 years experienced an increase in the prevalence of obesity (*P* < 0.05; Table S6 and S7, Fig. 2(g) and (i)).

## Discussion

This cross-sectional study showed that the BMI distribution and the prevalence of overweight and obesity among preschool children aged 4–6 years in the Rhine-Neckar County and the City of Heidelberg increased between 2013 and 2018. After adjusting for age, sex, and migration background, the significant increasing trend of overweight was only detected among 5-year old male children with migration backgrounds. The upward trend of obesity was observed only for male children aged 5 years and for female children aged 6 years with migration backgrounds. This is the first population-level study that uses a dataset with such a large sample of School Entry Health Examination data to illustrate the time trends of overweight and obesity among preschool children in Rhine-Neckar County and the City of Heidelberg.

Our study demonstrated that the BMI distribution was increasing, which was consistent with studies by Jena et al. [20] and Aachen et al. [21]. In recent decades, although the global trend of overweight and obesity among children has been increasing [22], previous studies reported that the prevalence of overweight and obesity among German preschool children showed a downward trend after 2004 [6, 7, 23]. From the second wave of the German Health Interview and Examination Survey for Children and Adolescents during 2014 to 2017, the national trend of overweight and obesity decreased from 13% in 2003 to 11% in 2017. In Europe, the trend also remained stable and even decreased from 30.3% to 25.6% during 1999 to 2016 [24]. Prevalence of other countries such as Australia (22.5% in 2006 to 21.6% in 2018) and Canada (30.7% in 2004 to 27.0% in 2013) also have the same trend as Europe. However, our study showed an opposite trend that the prevalence increased from 8.9% to 10.2% between 2013 and 2018, but remained a lower level than that of Germany and Europe. It has similar trends compared to China (8.8% in 2006 to 10.1% in 2014) [25] and the United States (7.2% in 2006 to 9.4% in 2018). The cause of this phenomenon may be that the trend of overweight and obesity varies widely in federal states [8], and different measurement or assessment methods of obesity [23] were conducted in different regions. Another reason could be that migration background is regarded as a potential health determinant for the prevalence of overweight and obesity [26]; Rhine-Neckar County and the City of Heidelberg belongs to Baden-Württemberg, where the proportion of people with an migration background accounted for the top three states in Germany [27]. Besides, previous studies provided clear evidence of a significant association between migrant status and less use of preventive services [28]. All of the above factors may lead to an increasing trend in the prevalence of overweight and obesity. Although the German government has emphasized controlling obesity in the National Sustainable Developmental Strategy in 2016 [29], it remains difficult to apply health promotion and prevention measures to young children, especially for preschool children. Decreased energy expenditure or increased energy intake was regarded to be the cause of increasing obesity [30]. Many countries have established recommendations regarding the amount of time children and adolescents should take part in physical activities [31]. Due to the different type of kindergartens and their possible space limitations, it is difficult to quantify the intensity and time of physical exercise available for preschool children. Although one study showed that preschool children were more physically active on forest playgrounds than on traditional playgrounds [32], the forest kindergartens only accounted for a small proportion in Rhine-Neckar County and the City of Heidelberg. Besides, preschool children usually spend a lot of time with parents, and parenting style greatly affects children’s behavior [33, 34], including different diet types and use of visual media, which may influence children’s weight and/or height. Previous studies which indicated the same correlation between parenting style and obesity also showed similar trends of overweight and obesity among preschool children in Bavaria from 1982 to 1997 [6] and in the City of Aachen from 1968–1999 [21]. Taken together, our findings indicate that the government should develop and propagate appropriate policy, if possible, adapted to the type of kindergarten, and help parents establish healthy parenting styles.

Although the general prevalence of overweight and obesity in Rhine-Neckar County and the City of Heidelberg showed an upward trend, after adjusting by age, sex, and migration background, the increasing trend of overweight and obesity was only observed among male children aged 5 years with migration backgrounds and female children aged 6 years with migration backgrounds. Migration background has already been regarded as an independent potential risk factor for overweight and obesity in earlier studies [35–38]. Previous studies [39, 40] have indicated an increasing trend of overweight and obesity in children from lower socioeconomic status compared with children from medium to higher socioeconomic status. Meanwhile, different culture and life style, dietary habits, physical activity, parents’ overweight, breast feeding, or media consumption all played crucial roles for childhood obesity in children with migration backgrounds [33, 34]. We also found discrepancies in trends of overweight and obesity after adjusting for age, which were similar to previous findings [5, 41, 42] which found that the prevalence of overweight and obesity increased with age. This disparity may be related to the period of adiposity rebound occurring between 4 and 7 years old [43, 44]. Previous studies showed that the capacity of infants and young children to control their food intake is affected by feeding pattern and eating control of mothers [45]. When children become more autonomous and more in control of their food intake when growing older, their ability to regulate energy balance may be damaged, which could lead to obesity [44]. Targeted intervention projects are warranted for vulnerable groups, especially for children aged 5–6 years, to optimize the preventive measures.

Our study used a large cross-sectional dataset collected between 2013 and 2018 from the successive surveillance of SEHE, which involved all preschool children in Rhine-Neckar County and the City of Heidelberg. Our sample size was representative of the general preschool population of Rhine-Neckar County and the City of Heidelberg and was able to display the secular trend. Moreover, after adjusting by sociodemographic factors, it realistically reflects the trend of overweight and obesity in this area, which could offers formative evidence to regional policy-makers for preventing and controlling the prevalence of overweight and obesity among vulnerable groups.

Our study has several limitations. Firstly, since this study was based on a cross sectional study design, it could not assess temporality. Secondly, the trend we evaluated was adjusted only by age, sex, and migration background, and other sociodemographic factors should be investigated in further research. Thirdly, our results only represented preschool children from Rhine-Neckar County and the City of Heidelberg, and it may be difficult to generalize this to other populations.

## Conclusions

In conclusion, the BMI and trend of overweight and obesity from 2013 to 2018 was increasing in Rhine-Neckar County and the City of Heidelberg, especially for male children aged 5 years and female children aged 6 years with migration background. Health promotion strategies including appropriate governmental policies, healthy parenting styles, public health service support, and health education for children with migration backgrounds will help address this challenge.

**Table 1:** Baseline characteristics of preschool children in Rhine-Neckar County and the City of Heidelberg

Characteristic	Number	Percentage (%)
Survey Year		
2013	5665	17.0
2014	5619	16.8
2015	4557	13.6
2016	5612	16.8
2017	5956	17.8
2018	5998	18.0
Total	33,407	100
Sex		
Male children	17,304	51.8
Female children	16,103	48.2
Age		
4	4062	12.2
5	25,078	75.0
6	4267	12.8
Migration background		
Non-migrant	16,436	49.2
Immigrant	16,971	50.8
Nutrition status		
Normal	29,605	88.5
Underweight	357	1.1
Overweight	2525	7.6
Obesity	920	2.8

## Supplementary Information

**Additional file 1:** **Table S1.** Mean BMI (kg/m^2^) in preschool children aged 4-6 years in Rhine-Neckar County and the City of Heidelberg, 2013-2018. **Table S2.** Trends in the prevalence of overweight in preschool children, 2013 to 2018. **Table S3.** Trends in the prevalence of overweight in male children, 2013 to 2018. **Table S4.** Trends in the prevalence of overweight in female children, 2013 to 2018. **Table S5. **Trends in the prevalence of obesity in preschool children, 2013 to 2018. **Table S6.** Trends in the prevalence of obesity in male children, 2013 to 2018. **Table S7.** Trends in the prevalence of obesity in female children, 2013 to 2018.

## Data Availability

The datasets used and/or analysed during the current study are available from the corresponding author on reasonable request.

## Abbreviation

SEHE: School Entry Health Examination
