# Supplementary material for: Trends of overweight and obesity among preschool children from 2013 to 2018: a cross-sectional study in Rhine-Neckar County and the City of Heidelberg, Germany
Source: BMC Public Health. 2022 May 11;22:941. doi: 10.1186/s12889-022-13302-w (PMC9092815; doi:10.1186/s12889-022-13302-w)
Supplement: Supplementary file 1 — Additional file 1: Table S1. Mean BMI (kg/m2) in preschool children aged 4-6 years in Rhine-Neckar County and the City of Heidelberg, 2013-2018. Table S2. Trends in the prevalence of overweight in preschool children, 2013 to 2018. Table S3. Trends in the prevalence of overweight in male children, 2013 to 2018. Table S4. Trends in the prevalence of overweight in female children, 2013 to 2018. Table S5. Trends in the prevalence of obesity in preschool children, 2013 to 2018. Table S6. Trends in the prevalence of obesity in male children, 2013 to 2018. Table S7. Trends in the prevalence of obesity in female children, 2013 to 2018. [file 12889_2022_13302_MOESM1_ESM.docx]

**SUPPLEMENTARY MATERIAL**

Table S1. Mean BMI (kg/m^2^) in preschool children aged 4-6 years in Rhine-Neckar County and the City of Heidelberg, 2013-2018

| Subgroups | Total | Aged 4 years | Aged 5 years | Aged 6 years |
| --- | --- | --- | --- | --- |
| Male children |  |  |  |  |
| 2013 | 15.5 | 15.8 | 15.5 | 15.5 |
| 2014 | 15.6 | 15.7 | 15.6 | 15.6 |
| 2015 | 15.7 | 15.7 | 15.6 | 16.0 |
| 2016 | 15.7 | 15.8 | 15.7 | 15.8 |
| 2017 | 15.8 | 15.8 | 15.8 | 16.0 |
| 2018 | 15.8 | 15.7 | 15.8 | 15.9 |
| 2013~2018 | 15.7 | 15.7 | 15.6 | 15.8 |
| *P* value for trend | 0.000* | 0.488 | 0.000* | 0.000* |
| Female children |  |  |  |  |
| 2013 | 15.4 | 15.4 | 15.3 | 15.6 |
| 2014 | 15.5 | 15.5 | 15.5 | 15.4 |
| 2015 | 15.6 | 15.6 | 15.6 | 15.8 |
| 2016 | 15.6 | 15.8 | 15.6 | 15.7 |
| 2017 | 15.6 | 15.8 | 15.6 | 15.8 |
| 2018 | 15.6 | 15.6 | 15.6 | 15.8 |
| 2013~2018 | 15.6 | 15.6 | 15.5 | 15.7 |
| *P* value for trend | 0.000* | 0.024* | 0.000* | 0.003* |

*P* value for trend was calculated by general linear model.

**P*<0.05

Table S2. Trends in the prevalence of overweight in preschool children, 2013 to 2018

|  | Total (%) | Aged 4 years (%) | Aged 5 years (%) | Aged 6 years (%) |
| --- | --- | --- | --- | --- |
| **Total** | 2525 (7.9) | 125(3.1) | 1799(7.1) | 601 (14.1) |
| 2013 | 381 (6.7) | 21 (2.9) | 269 (6.3) | 91 (12.7) |
| 2014 | 420 (7.4) | 21 (3.1) | 299 (7.2) | 100 (12.7) |
| 2015 | 321 (7.0) | 16 (2.6) | 217 (6.4) | 88 (15.5) |
| 2016 | 458 (8.2) | 20 (3.7) | 313 (7.4) | 125 (14.4) |
| 2017 | 509 (8.5) | 19 (2.9) | 372 (8.1) | 118 (15.3) |
| 2018 | 436 (7.2) | 28 (3.1) | 329 (7.2) | 79 (13.7) |
| *P* value for trend | 0.018* | 0.864 | 0.008* | 0.167 |

*P* value for trend were calculated by Logistic regression models using the survey year as a continuous variable.

**P*<0.05

Table S3. Trends in the prevalence of overweight in male children, 2013 to 2018

| Male children | Total Male children | Aged 4 years (%) | Aged 5 years (%) | Aged 6 years (%) |
| --- | --- | --- | --- | --- |
| **Total** | 1363 (7.9) | 75 (3.6) | 961 (7.4) | 51 (14.6) |
| 2013 | 209 (7.1) | 13 (3.6) | 145 (6.5) | 55 (13.4) |
| 2014 | 226 (7.9) | 11 (3.3) | 160 (7.7) | 42 (13.2) |
| 2015 | 169 (7.1) | 9 (2.9) | 118 (6.6) | 65 (14.4) |
| 2016 | 232 (7.9) | 13 (4.7) | 154 (7.0) | 66 (14.7) |
| 2017 | 276 (8.9) | 11 (3.4) | 199 (8.4) | 48 (16.2) |
| 2018 | 251 (8.2) | 18 (4.0) | 185 (8.0) | 299 (15.6) |
| *P* value for trend | 0.024* | 0.633 | 0.030* | 0.133 |
| **With migration background** |  |  |  |  |
| 2013 | 90 (6.0) | 5 (2.4) | 61 (5.4) | 24 (15.8) |
| 2014 | 91 (6.2) | 9 (4.4) | 55 (5.2) | 27 (14.2) |
| 2015 | 65 (5.5) | 5 (2.8) | 47 (5.3) | 13 (12.9) |
| 2016 | 107 (7.0) | 10 (6.9) | 68 (5.8) | 29 (13.7) |
| 2017 | 119 (7.6) | 2 (1.3) | 91 (7.3) | 26 (16.1) |
| 2018 | 111 (7.0) | 8 (3.3) | 80 (6.7) | 23 (15.6) |
| *P* value for trend | 0.051 | 0.931 | 0.018* | 0.798 |
| **With non-migration background** |  |  |  |  |
| 2013 | 119 (8.1) | 8 (5.0) | 84 (7.7) | 27 (11.8) |
| 2014 | 135 (9.8) | 2 (1.6) | 105 (10.3) | 28 (12.3) |
| 2015 | 104 (8.6) | 4 (3.1) | 71 (8.0) | 29 (15.3) |
| 2016 | 125 (9.0) | 3 (2.2) | 86 (8.3) | 36 (15.6) |
| 2017 | 157 (10.1) | 9 (5.4) | 108 (9.5) | 40 (16.3) |
| 2018 | 140 (9.3) | 10 (4.8) | 105 (9.3) | 25 (17.5) |
| *P* value for trend | 0.187 | 0.461 | 0.364 | 0.072 |

*P* value for trend were calculated by Logistic regression models using the survey year as a continuous variable.

**P*<0.05

Table S4. Trends in the prevalence of overweight in female children, 2013 to 2018

|  | Total Female children | Aged 4 years (%) | Aged 5 years (%) | Aged 6 years (%) |
| --- | --- | --- | --- | --- |
| **Total** | 1162 (7.2) | 274 (2.5) | 838 (6.9) | 274 (13.6) |
| 2013 | 172(6.3) | 40 (12.0) | 124 (6.1) | 40 (12.1) |
| 2014 | 194 (7.0) | 45 (12.3) | 139 (6.7) | 45 (12.3) |
| 2015 | 152 (7.0) | 46 (16.8) | 99 (6.2) | 46 (16.8) |
| 2016 | 226 (8.4) | 60 (14.2) | 159 (7.9) | 60 (14.2) |
| 2017 | 233 (8.2) | 52 (14.4) | 173 (8.0) | 52 (14.4) |
| 2018 | 185 (6.3) | 31 (11.6) | 144 (6.5) | 31 (11.6) |
| *P* value for trend | 0.274 | 0.769 | 0.118 | 0.682 |
| **Migration background** |  |  |  |  |
| 2013 | 67 (4.9) | 2 (1.2) | 50 (4.7) | 15 (10.9) |
| 2014 | 89 (6.2) | 5 (2.8) | 70 (6.4) | 14 (7.9) |
| 2015 | 51 (4.8) | 4 (2.4) | 33 (4.2) | 14 (12.7) |
| 2016 | 102 (7.4) | 4 (3.1) | 72 (6.8) | 26 (13.1) |
| 2017 | 82 (5.7) | 2 (1.2) | 65 (5.7) | 15 (10.5) |
| 2018 | 95 (6.4) | 6 (2.7) | 73 (6.4) | 16 (12.5) |
| *P* value for trend | 0.119 | 0.706 | 0.149 | 0.326 |
| **Non-migration background** |  |  |  |  |
| 2013 | 105 (7.9) | 6 (3.6) | 74 (7.7) | 25 (12.9) |
| 2014 | 105 (7.9) | 5 (3.2) | 69 (7.0) | 31 (16.3) |
| 2015 | 101 (9.0) | 3 (2.3) | 66 (8.0) | 32 (19.5) |
| 2016 | 124 (9.5) | 3 (2.3) | 87 (9.1) | 34 (17.1) |
| 2017 | 151 (10.8) | 6 (4.0) | 108 (10.5) | 37 (16.3) |
| 2018 | 90 (6.3) | 4 (1.8) | 71 (6.6) | 15 (10.8) |
| *P* value for trend | 0.928 | 0.457 | 0.421 | 0.811 |

*P* value for trend were calculated by Logistic regression models using the survey year as a continuous variable.

**P*<0.05

Table S5. Trends in the prevalence of obesity in preschool children, 2013 to 2018

|  | Total (%) | Aged 4 years (%) | Aged 5 years (%) | Aged 6 years (%) |
| --- | --- | --- | --- | --- |
|  |  |  |  |  |
| **Total** | 920 (2.7) | 35 (0.8) | 629 (2.5) | 256 (6.0) |
| 2013 | 129 (2.2) | 8 (1.1) | 84 (2.0) | 37 (5.2) |
| 2014 | 142 (2.5) | 9 (1.3) | 106 (2.5) | 27 (3.4) |
| 2015 | 137 (3.0) | 4 (0.6) | 91 (2.6) | 42 (7.4) |
| 2016 | 175 (3.1) | 7 (1.3) | 113 (2.6) | 55 (6.3) |
| 2017 | 155 (2.6) | 5 (0.7) | 99 (2.1) | 51 (6.6) |
| 2018 | 182 (3.0) | 2 (0.2) | 136 (3.0) | 44 (7.6) |
| *P* value for trend | 0.019* | 0.033* | 0.034* | 0.004* |

*P* value for trend were calculated by Logistic regression models using the survey year as a continuous variable.

**P*<0.05

Table S6. Trends in the prevalence of obesity in male children, 2013 to 2018

|  | Total (%) Aged 4 years (%) Aged 5 years(%) No. (%) | | | |
| --- | --- | --- | --- | --- |
|  |  |  |  | Aged 6 years |
| **Total** | 556 (3.2) | 22 (1.1) | 371 (2.9) | 163 (7.3) |
| 2013 | 79 (2.7) | 7 (1.9) | 51 (2.3) | 21 (5.5) |
| 2014 | 87 (3.1) | 6 (1.8) | 62 (3.0) | 19 (4.5) |
| 2015 | 78 (3.3) | 3 (1.0) | 45 (2.5) | 30 (10.3) |
| 2016 | 108 (3.7) | 2 (0.7) | 71 (3.2) | 35 (7.9) |
| 2017 | 102 (3.3) | 3 (0.9) | 65 (2.7) | 34 (8.4) |
| 2018 | 102 (3.3) | 1 (0.2) | 77 (3.3) | 24 (7.8) |
| *P* value for trend | 0.093 | 0.062 | 0.074 | 0.035* |
| **Migration background** |  |  |  |  |
| 2013 | 29 (1.9) | 3 (1.5) | 17 (1.5) | 9 (5.9) |
| 2014 | 25 (1.7) | 2 (1.0) | 19 (1.8) | 4 (2.1) |
| 2015 | 19 (1.6) | 1 (0.6) | 10 (1.1) | 8 (7.9) |
| 2016 | 33 (2.2) | 1 (0.7) | 22 (1.9) | 10 (4.7) |
| 2017 | 29 (1.9) | 0 (0) | 19 (1.5) | 10 (6.2) |
| 2018 | 41 (2.6) | 0 (0) | 36 (3.0) | 5 (3.4) |
| *P* value for trend | 0.149 | 0.047 | 0.033* | 0.489 |
| **Non-migration background** |  |  |  |  |
| 2013 | 50 (3.4) | 4 (2.5) | 34 (3.1) | 12 (5.3) |
| 2014 | 62 (4.5) | 4 (3.1) | 43 (4.2) | 15 (6.6) |
| 2015 | 59 (4.9) | 2 (1.5) | 35 (3.9) | 22 (11.6) |
| 2016 | 75 (5.4) | 1 (0.7) | 49 (4.8) | 25 (10.8) |
| 2017 | 73 (4.7) | 3 (1.8) | 46 (4.1) | 24 (9.8) |
| 2018 | 61 (4.1) | 1 (0.5) | 41 (3.6) | 19 (11.8) |
|  | 0.274 | 0.085 | 0.525 | 0.059 |

*P* value for trend were calculated by Logistic regression models using the survey year as a continuous variable.

**P*<0.05

Table S7. Trends in the prevalence of obesity in female children, 2013 to 2018

|  | No. (%) of female obesity | | | |
| --- | --- | --- | --- | --- |
|  | Total | Aged 4 years | Aged 5 years | Aged 6 years |
| **Total** | 364 (2.3) | 13 (0.7) | 258 (2.1) | 93 (4.6) |
| 2013 | 50 (1.9) | 1 (0.3) | 33 (1.6) | 16 (4.8) |
| 2014 | 55 (2.0) | 3 (0.9) | 44 (2.1) | 8 (2.2) |
| 2015 | 59 (2.7) | 1 (0.3) | 46 (2.9) | 12 (4.4) |
| 2016 | 67 (2.5) | 5 (1.9) | 42 (2.1) | 20 (4.7) |
| 2017 | 53 (1.9) | 2 (0.6) | 34 (1.6) | 17 (4.7) |
| 2018 | 80 (2.7) | 1 (0.2) | 59 (2.7) | 20 (7.5) |
| *P* value for trend | 0.877 | 0.472 | 0.246 | 0.043* |
| **Migration background** |  |  |  |  |
| 2013 | 19 (1.4) | 1 (0.6) | 13 (1.2) | 5 (3.6) |
| 2014 | 18 (1.3) | 2 (1.1) | 14 (1.3) | 2 (1.1) |
| 2015 | 22 (2.1) | 0 (0) | 17 (2.2) | 5 (4.5) |
| 2016 | 26 (1.9) | 3 (2.3) | 15 (1.4) | 8 (4.0) |
| 2017 | 25 (1.7) | 0 (0) | 16 (1.4) | 9 (6.3) |
| 2018 | 27 (1.8) | 0 (0) | 19 (1.7) | 8 (6.3) |
| *P* value for trend | 0.208 | 0.306 | 0.489 | 0.040* |
| **Non-migration background** |  |  |  |  |
| 2013 | 31 (2.3) | 0 (0) | 20 (2.1) | 11 (5.7) |
| 2014 | 37 (2.8) | 1 (0.6) | 30 (3.0) | 6 (3.2) |
| 2015 | 37 (3.3) | 1 (0.8) | 29 (3.5) | 7 (4.3) |
| 2016 | 41 (3.1) | 2 (1.5) | 27 (2.8) | 12 (5.4) |
| 2017 | 28 (2.0) | 2 (1.3) | 18 (1.7) | 8 (3.7) |
| 2018 | 53 (3.7) | 1 (0.4) | 40 (3.7) | 12 (8.6) |
| *P* value for trend | 0.252 | 0.467 | 0.359 | 0.389 |

*P* value for trend were calculated by Logistic regression models using the survey year as a continuous variable.

**P*<0.05
